# Effects of Biodegradation on the Structure and Properties of Windmill Palm (*Trachycarpus fortunei*) Fibers Using Different Chemical Treatments

**DOI:** 10.3390/ma10050514

**Published:** 2017-05-09

**Authors:** Changjie Chen, Weiwei Yin, Guicui Chen, Guangxiang Sun, Guohe Wang

**Affiliations:** 1 College of Textile and Clothing Engineering, Soochow University, Suzhou 215006, China; ccjsd2013@163.com (C.C.); 20154215008@stu.suda.edu.cn (W.Y.); 20154015008@stu.suda.edu.cn (G.C.); 20145215010@stu.suda.edu.cn (G.S.); 2 Nantong Textile & Silk Industrial Technology Research Institute, Nantong 226108, China; 3 Jiangsu Research and Development Center of the Ecological Textile Engineering and Technology, Yancheng Institute of Industry Technology, Yancheng 224005, China

**Keywords:** windmill palm fiber, alkali-treated fiber, bleached fiber, biodegradation, mechanical property

## Abstract

In this work, windmill palm fiber (WPF), alkali-treated fiber (AF) without hemicellulose and bleached fiber (BF) without lignin were prepared and buried in soil for 30, 60 and 90 days. The surface morphology, chemical composition, crystallinity degree, mechanical properties, and residual mass rate of the samples, before and after biodegradation, were investigated. According to the results, soil burial degradation can remove the parenchyma cells and silica-bodies of WPF and deplete droplets containing the lignin of alkali-treated fiber after it has been buried for 30 days (AF30), and degradation of the single fiber cell wall of bleached fiber after it has been buried for 30 days (BF30). Buried in natural soil, lignin has a slower degradation rate than that of hemicellulose. WPF showed no significant differences in tensile strength after burial in soil for 90 days, because of the integrity fiber structure decreased the biodegradation. The most serious decrease, about 43%, in tensile strength occurred in AF after it had been buried for 90 days (BF90). This basic knowledge may be helpful for windmill palm fiber applications, especially for biodegradable composites.

## 1. Introduction

Cellulose, a dominant component in the vast majority of plant forms, which has an annual production that is estimated to be around 1.0 × 10^11^~1.0 × 10^12^ t [1,2,3], is a promising resource. In particular, annually renewable agricultural residues represent an abundant, inexpensive, and readily-available source of renewable lignocellulosic biomass [4]. Increased attentions have been given to the production of novel materials for environmentally-friendly industrial use, post chemical modification [5]. Sodium chlorite (NaClO_2_) treatment is a common method for the bleaching of fibers. The oxidation reaction can form chlorine dioxide (ClO_2_), which reacts with lignin constituents, and, thus, lignin is removed from fiber [6,7,8]. Alkali treatment is also a useful method of eliminating amorphous hemicellulose in order to improve the mechanical properties of lignocellulose fiber [9,10,11].

Composite materials are one of the most significant inventions of the material sciences, which can be used in many fields, and have high-quality and low-cost applications [12]. The due criticism of synthetic reinforcement of composites is that they are not easily biodegradable. In recent years, there has been tremendous growth in the study and development of natural fiber-reinforced composite materials [13]. Among reinforcing fibers, palm fiber appears to be a promising material because it is relatively inexpensive and abundantly available. Windmill palm fiber is part of the palm fiber family and has abundant lignocellulose materials. Presently, in China, most of the abundant, inexpensive, and readily-available windmill palm leaf sheaths are treated as waste or as fuel, which creates environmental-pollution problems. Some of the leaf sheaths are used to make mattress and other products of low additional value and low efficiency, such as whisk brooms brushes and ropes [14]. However, windmill palm fiber has a tensile strength that is equal to that of surge palm [15], and has the potential to be used as a reinforcement fiber to make biodegradation composites. Studies on biodegradation behavior are important for the application of windmill palm fiber as a reinforcement fiber for biodegradable composites in environmental aging.

This paper focuses on the properties of windmill palm fibers (WPF), alkali-treated windmill palm fibers (AF), and bleached windmill palm fibers (BF), before and after soil burial degradation. The effects of biodegradation on morphology structure, chemical composition, crystallinity degree, and the mechanical properties of the fibers, with and without chemical treatment, are studied. The goal of this study is to provide background information that will hopefully be of use in future fiber applications, especially in biodegradable composites.

## 2. Materials and Methods

### 2.1. Materials 

Untreated windmill palm fiber was drawn off of a mesh that was obtained from the Yuanmu Company in Hubei province, China. The bleached fiber was prepared using a 0.007 M NaClO_2_ solution and 0.022 M CH_3_COOH with a water bath of 1:50 (fiber-to-liquor ratio) for 2 h at 80 °C for 3 times [16]. The raw windmill palm fiber was soaked in 1.5 M, 2 M and 2.5 M NaOH solutions, one at a time, in a water bath of 1:50. The temperature was maintained at 60 °C for 2 h to prepare the alkali-treated fiber [15].

Untreated WPF, after biodegradation in soil for 30, 60, and 90 days, were WPF30, WPF60 and WPF90, respectively; alkali-treated fiber, after biodegradation in soil for 30, 60 and 90 days, were AF30, AF60 and AF90, respectively; and bleached fiber, after biodegradation in soil for 30, 60, and 90 days, were BF30, BF60 and BF90, respectively.

### 2.2. Methods

#### 2.2.1. Morphology Study

The surface morphologies of the WPF samples, with and without chemical treatment, before and after burial in soil, were examined using scanning electron microscope (SEM, TM3030, Hitachi, Tokyo, Japan). For SEM analyses, all samples were sputter coated with a thin layer of gold and observed using SEM at an accelerating voltage of up to 1.5 kV.

#### 2.2.2. Fourier Transforms Infrared Spectroscopy (FTIR)

Windmill palm fiber samples, before (WPF, AF, and BF) and after soil degradation (WPF30, WPF60, WPF90, AF30, AF60, AF90, BF30, BF60, and BF90), were subjected to FTIR analyses (Perki-Elmer, Waltham, MA, USA). The spectra were obtained for wave numbers ranging between 4000 and 400 cm^−1^.

#### 2.2.3. X-ray Diffraction Analysis

X-ray diffractograms of windmill palm fiber, before and after degradation, were obtained using an X-ray powder diffractometer (Xpert-Pro MPD, PANalytical B.V., Almelo, The Netherlands). The test was operated at room temperature, using a Cu-Kα source (λ = 1.54 nm) for 2θ between 5° and 45°. The crystallinity index was calculated according to the following equation: Cr*I* % = [(*I*_002_ − *I*_am_)/*I*_002_] × 100. Where *I*_002_ shows that the peak intensity of the main crystalline plane diffraction was located at about 22°, and *I*_am_ shows that the intensity of the amorphous fraction materials was observed at about 18°.

#### 2.2.4. Mechanical Testing

The tensile properties of windmill palm fiber samples were measured using a universal testing machine (Instron 5967, Instron, Norwood, MA, USA). The load capacity was 500 N and the cross-head speed was 0.05 mm/s; gauge lengths of 20 mm, 30 mm, and 40 mm were used. For each sample, the tensile strength of 5 fibers was measured for each gauge length in order to analyze the tensile modulus, tensile strength, and elongation at break, according to the ASTM C1557 “Standard Test Method for Tensile Strength and Young’s Modulus of Fibers” [17].

#### 2.2.5. Soil Burial Degradation

Three samples (2 g) of each kind of fiber were buried in soil. The biodegradability of the samples, buried in the soil of a garden, were assessed by measuring the rate of residual mass. After drying and weighing, samples were wrapped with 200 mesh and were buried in garden soil at a depth of 20 cm. The pH of the soil was 8.1, and the particle size distribution of the clay soil was between 122 nm and 1110 nm. The moisture content of the soil was 15.83%. The buried samples were carefully withdrawn after being buried for 30, 60, and 90 days, respectively. Then, the samples were washed with distilled water and dried to a constant weight, at 50 °C in a vacuum oven. The rate of residual mass (%) was calculated using the formula: Rate of residual mass *%* = *W_t_/W*_o_ × 100. Where *W*_o_ is the weight of the sample before biodegradation, and *W_t_* is the weight of the sample after biodegradation (at time *t*).

## 3. Results and Discussion

### 3.1. Analysis of Windmill Palm Fiber Morphology Structure

**Figure 1:**
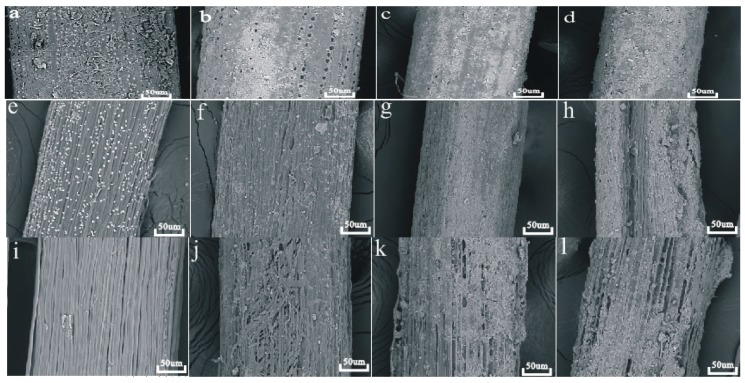
SEM images of the surface morphologies of windmill palm fibers, before and after 30-, 60-, and 90-day soil burial. Untreated windmill palm fiber (**a**); after biodegradation in soil for 30 days (WPF30) (**b**); 60 days (WPF60) (**c**); and 90 days (WPF90) (**d**). Alkali-treated fibers (AF) (**e**); after biodegradation in soil for 30 days (AF30) (**f**); 60 days (AF60) (**g**); and 90 days (AF90) (**h**). Bleached fibers (BF) (**i**); after biodegradation in soil for 30 days (BF30) (**j**); 60 days (BF60) (**k**); and 90 days (BF90) (**l**).

SEM micrographs of the surface of windmill palm fiber samples, before and after being buried in soil for different times, are shown in Figure 1. The attached parenchyma cells of the WPFs had coarse surfaces (Figure 1a). Under the parenchyma cells were silica-bodies [14]. After 30 days, the degradation removed the parenchyma cells and silica-bodies. Only conical-shaped circles remained (Figure 1b). Inorganic substances from the soil were physically attached to the surface of windmill palm fiber after 60 days (Figure 1c). This situation worsened as the time went on. After 90 days, the fiber surface was coated with a thick layer of inorganic substances (Figure 1d).

After alkali treatment, a large number of droplets, containing lignin, were observed on the alkali-treated windmill palm fiber surface, as is shown in Figure 1e [15]. These droplets almost disappeared after the fiber was buried in soil for 30 days (Figure 1f). As with the raw windmill palm fiber, a thin layer of inorganic substances covered the fiber surface after 60 days (Figure 1g) and it was thicker after 90 days (Figure 1h). 

It can be seen in Figure 1i that the bleached windmill palm fiber had a striated surface. This is the result of the delignification process exposing the single fibers, and the removal of attached parenchyma cells, silica-bodies, and most of the hemicellulose. After 30 days, the cell wall of the single fibers had been partially degraded (Figure 1j). Several grooves were found on the surface of windmill palm fiber indicating further degradation of the single fiber cell wall. After 90 days, a large number of grooves showed the serious degradation of palm fiber (Figure 1l).

### 3.2. FTIR Structural Analyses

**Figure 2:**
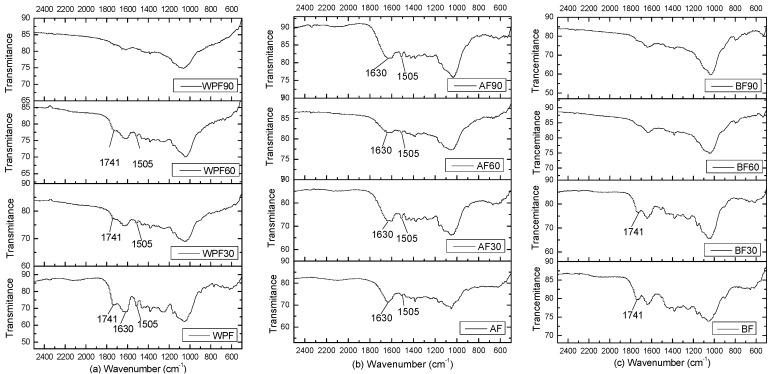
Fourier Transforms Infrared (FT-IR) spectra of (**a**) windmill palm fiber (**b**) alkali-treated fiber and (**c**) bleached fiber before and after biodegradation.

The changes in the functional group that occurred in the WPF, before and after soil degradation, are shown in Figure 2. The peak at about 1740 cm^−1^ indicated the presence of C=O stretching in the acetyl groups of hemicelluloses [18,19]. The peak disappeared at 1741 cm^−1^ for AF, which indicated the removal of hemicelluloses by the alkali treatment. Likewise, samples AF30, AF60 and AF90 were free of hemicellulose. The absence of the 1741 cm^−1^ band of BF and WPF can be seen after soil degradation for 60 and 90 days, respectively. This indicates the almost total degradation of hemicellulose in windmill palm fibers. 

The band at 1505 cm^−1^ of BF was gradually reduced with a bleach treatment, which indicated the reduction of lignin content [20,21,22]. The absence of the FTIR spectra band of WPF90 indicated that no lignin was present in the sample; the AF90 sample still had a clear band at 1505 cm^−1^. This indicates the slower degradation rate of lignin compared to hemicellulose (when buried in soil). The peak at 1630 cm^−1^ was due to the characteristic axial vibrations of the hydroxyl group of cellulose [23]. Reduction of this peak intensity is indicative of cellulose degradation. Our results show a complete disappearance of the 1630 cm^−1^ peak for the BF sample, while it is still visible for the WPF and AF samples. This suggests a faster degradation of cellulose for the bleached sample.

### 3.3. X-ray Diffraction and Crystallinity Measurements

**Figure 3:**
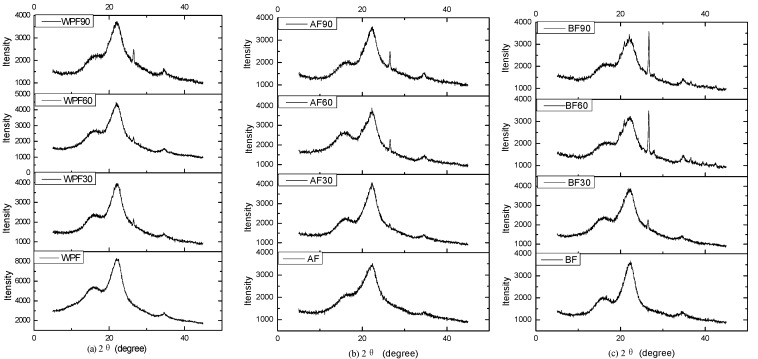
X-ray diffractograms for (**a**) windmill palm fiber (**b**) alkali-treated fiber and (**c**) bleached fiber before and after soil burial.

Figure 3 shows the typical XRD diffractogram patterns of cellulose I. The spectra of windmill palm fiber, before and after soil degradation, showed two major reflections corresponding to the 2θ values of 18° (amorphous phase) and 22° (crystallographic plane of cellulose I) [24]. AF60, BF60, WPF90, AF90, and BF90 showed narrow diffractions peaks, attributed to an unknown contamination. The longer the burial time, the more intense the narrow peak. The peak about 26° may be the inorganic substances from the soil that attached to the fiber surface. Guimarães observed these narrow peaks at 26° in banana fiber, and correlated them to an inorganic substance [25]. Pereira also believed that the narrow peaks at about 26° correlated to the salts of inorganic components, such as potassium, chloride, calcium, and phosphor [26].

The crystallinity index of each sample was calculated, and the results are shown in Table 1. The crystallinity index of WPF was 34%; this value increased to 50% after bleach treatment. After being buried in soil for 30 days, the crystallinity of both WPF30 and AF30 increased due to the degradation of amorphous materials. However, the crystallinity of BF30 decreased to 43% and the degradation of cellulose may be the reason for these decreases, which would be consistent with the obtained results (Figure 1j). The decrease in crystallinity continues for all the samples in soil for 60 days, but does not seem to continue beyond that.

### 3.4. Tensile Property Analyses

**Figure 4:**
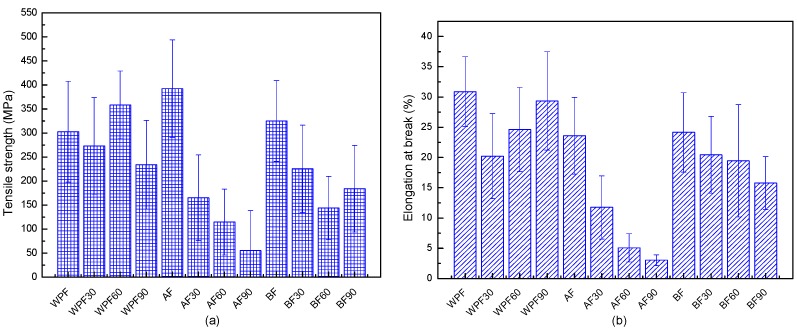
(**a**) Tensile strength and (**b**) elongation at break of windmill palm fiber with and without chemical treatment before and after soil burial.

Figure 4 shows the tensile properties of windmill palm fiber, before and after soil degradation. The mechanical properties of windmill palm fiber treated using alkali or bleach treatment have been discussed in previous work [15]. The tensile strength of WPF increased by 30% after the alkali treatment, which is probably due to the removal of hemicellulose and some impurities and the decrease of the fiber diameter. The tensile strength of AF30 and BF30 decreased the most relative to AF60, BF60, AF90, and BF90. 

The tensile strength, as well as the elongation at break, showed a linear decrease of about 50 MPa and 5% per 30 days for AF30, AF60, and AF90. The tensile strength and elongation at break for the bleached samples after soil burial for 90 days decreased by around 43% and 38% compared with those of BF. The tensile modulus for windmill palm fiber, alkali-treated fiber, and bleached fiber, before and after biodegradation, showed little difference. Additionally, the results were about 3 GPa, 8 GPa and 4 GPa, respectively.

In order to improve the mechanical properties of wood plastic composites, lignocellulose fiber has been treated by different chemical treatments (especially alkalize treatment) [27,28]. Our results showed that alkalize treatment can improve the tensile property, but decrease the durable property at the same time. For the seriously decrease of tensile strength at the first 30 days.

The analysis results for the mean tensile strength and elongation at break for windmill palm fiber, before and after degradation, are shown in Table 2. After the fiber was buried in soil for 30 days, only the alkali treated fiber experienced a significant tensile strength loss. This may indicate serious structural damage to the alkali-treated fiber. WPF and WPF30, as well as the BF and BF30 showed no significant differences. Elongation at break for WPF and WPF30, as well as AF and AF30, had significant differences. A significant difference for bleached fiber can be seen until 60 days. The strain increases with the decrease in micro-fibril angle. The decrease in the elongation at break may indicate that soil burial increases the micro-fibril angle. 

### 3.5. Mass Loss in Soil

**Figure 5:**
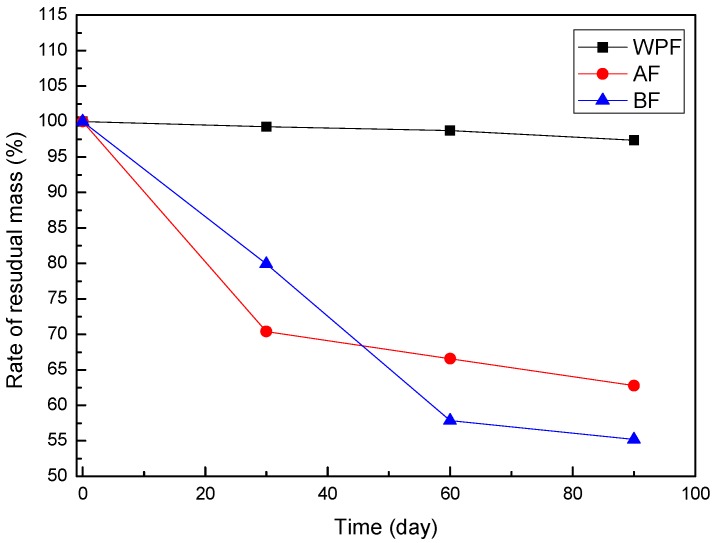
Rate of residual mass of windmill palm fiber (WPF), alkali-treated fiber (AF), and bleached fiber (BF), before and after biodegradation.

Figure 5 shows the residual mass rate of windmill palm fiber samples, before and after being buried in soil for different lengths of time. In general, the biodegradation of polymers may involve a number of different steps, including deterioration by decomposing organisms and/or abiotic factors, depolymerization with the reduction in molecular weight, and assimilation [29]. Almost all periods showed mass loss; WPF showed no observable changes in terms of mass. Almost 97% of the residues remained at 90 days for the untreated windmill palm fiber, from the garden soil burying experiment, suggesting that WPF possess a fairly good durable property. After 30 days of soil burial, AF30 and BF30 exhibited the most severe signs of degradation, with a significant mass loss of about 30% and 20%, respectively. This was in good agreement with the tensile property observations for the most severe signs of tensile strength loss, which also occurs in the first 30 days for AF30 (58%) and BF30 (31%). 

Note that weight loss shows an approximately linear relation with degradation time for both AF and BF, with an average degradation rate about 0.4%/day and 0.5%/day, respectively. It was observed that the residual mass of AF was higher than that of BF at the 60- and 90-day points. The structural complexity of lignin, its high molecular weight, and its insolubility make its degradation very difficult [30]. The AF with lignin has a worse biodegradation than that of BF. The residual mass of BF90 is about 55%, indicating a preferential biodegradation property. 

## 4. Conclusions

In this study, the surface morphology, chemical composition, crystallinity degree, mechanical properties, and residual mass rate of samples, before and after biodegradation, were investigated. The soil burial degradation removed parenchyma cells and silica-bodies from WPF, as well as droplets containing lignin, from the surface of AF in the first 30 days. In a natural soil environment, hemicellulose showed a faster degradation rate relative to lignin (AF90 still contained lignin, while BF60 was nearly without hemicellulose). This was especially true in the absence of lignin (Disappearance of hemicellulose in BF60 compare to WPF60), therefore, Lignin acted to protect the hemicellulose. 

The crystallinity index of BF increased to 50%, as the bleach treatment removed the hemicellulose. The crystallinity degree of WPF30 and AF30 increased to 42% and 47%, respectively, for the degradation of amorphous materials; however, the crystallinity degree of BF30 decreased to 43% due to the decrease in cellulose. During the last 30 days (from 60 to 90 days), a lower loss of mass as well as the relatively stable crystallinity degree indicated a slower degradation of fiber. The coated thick layer of inorganic substances may decrease biodegradation.

The tensile strength and elongation at break for the bleached samples after soil burial for 90 days decreased by about 43% and 38% compared with those of BF. The AF30 and BF30 samples exhibited the most severe signs of degradation, with a significant mass loss of about 30% and 20%, which was in accordance with the severe signs of tensile strength loss for AF30 (58%) and BF30 (31%). Overall the results were showing that the integrity of the fiber is important for increase biodegradation as appearance of grooves, loss of mass and mechanical properties decreases only happen with bleach and alkalize treatment.

## Figures and Tables

**Table 1:** Crystallinity of windmill palm fiber, before and after degradation.

Samples	Peak Position (2θ)	*I*_002_ (cps)	Peak Position (2θ)	*I*am (cps)	Cr*I* (%)
WPF	21.61	3148	18.10	2093	34
WPF30	22.06	3894	18.47	2276	42
WPF60	22.09	4342	18.18	2549	41
WPF90	22.09	3666	17.94	2169	41
AF	22.33	3434	17.82	2065	40
AF30	22.37	4026	18.27	2118	47
AF60	22.29	3710	18.50	2315	38
AF90	22.26	3458	17.78	2157	38
BF	22.34	3574	18.46	1801	50
BF30	22.26	3802	18.19	2184	43
BF60	22.25	3125	18.62	1962	37
BF90	22.17	3240	18.58	2019	38

**Table 2:** Analyses of the mean tensile strength and elongation at break for windmill palm fiber, before and after degradation.

**Tensile Strength**								
**Samples**	**N**	**Subset of Alpha = 0.05**						
		1	2	3	4	5	6	7
AF90	15	88.5742						
AF60	15	114.7384	114.7384					
BF60	15	163.7056	163.7056	163.7056				
AF30	15	165.1365	165.1365	165.1365				
BF90	15		183.9735	183.9735				
BF30	15			225.1725	225.1725			
WPF90	15			233.801	233.801			
WPF30	15				273.1758	273.1758		
WPF	15				291.5799	291.5799	291.5799	
BF	15					328.2119	328.2119	328.2119
WPF60	15						358.308	358.308
AF	15							390.5304
Sig.		0.059	0.089	0.093	0.103	0.163	0.09	0.113
**Elongation at Break**								
AF90	15	3.0213						
AF60	15	5.0593						
AF30	15		11.748					
BF90	15		15.7653	15.7653				
BF60	15			19.452				
WPF30	15			20.2353	20.2353			
BF30	15			20.442	20.442			
WPF60	15				24.6113	24.6113		
AF	15				24.8583	24.8583		
BF	15				24.9913	24.9913		
WPF90	15					29.3487	29.3487	
WPF	15						31.0407	
Sig.		0.374	0.081	0.062	0.064	0.059	0.46	
